# Efficient single step chromatographic purification of recombinant human antithrombin (rhAT) from *Saccharomyces cerevisiae*

**DOI:** 10.1007/s13205-016-0412-z

**Published:** 2016-05-17

**Authors:** Maheswara Reddy Mallu, Sandeep Vemula, Srinivasa Reddy Ronda

**Affiliations:** Centre for Bioprocess Technology, Department of Biotechnology, KLEF University, Green Fields, Vaddeswaram, Guntur, Andhra Pradesh 522 502 India

**Keywords:** Antithrombin, Cross flow filtration, Chromatographic purification, *Saccharomyces cerevisiae*, Ion exchange and size exclusion

## Abstract

Antithrombin (AT) is a glycoprotein that inactivates the several physiological target enzymes of coagulation system. The effect of purification strategies plays a crucial role in getting maximum recovery of yield, purity and biological activity of recombinant human antithrombin (rhAT). In the present work, the task of purifying rhAT from *Saccharomyces cerevisiae* BY4741 has been carried out using two different approaches such as cross flow filtration (CFF) system and chromatography methods. In the first approach, the protein was concentrated and partially purified through CFF to achieve maximum recovery yield and purity of 87 and 94 %, respectively. In the second approach, purification involved a single step chromatography with various types of ion exchange and size exclusion resins to analyze the maximum rhAT recovery yield and purity. From the experimental results, it has been observed that the size exclusion chromatography (SEC) technique with Superose 12 matrix was suitable for the purification of rhAT and achieved the maximum recovery yield and purity of 51 and 97 %, respectively. Further, to acquire a high recovery yield and purity of rhAT, the effect of various chromatographic conditions such as mobile phase, mobile phase pH, flow rate, sample volume and sample concentration were also investigated. Under the optimal chromatographic conditions, rhAT was significantly recovered and purified in a single step with maximum recovery yield, purity and biological activity of 67, 99 % and 410 IU/L, respectively. Based on these investigations, it was concluded that SEC with Superose 12 matrix was a more suitable and a potential method for the purification of rhAT.

## Introduction

Antithrombin (AT) is a plasma glycoprotein (432 amino acids) and a member of serine protease inhibitor (SERPIN) family, which is an important physiological controller of various clotting factors, including thrombin, factor IXa, Xa, XIa, and XIIa (Rosenberg and Damus 1973; Franzen et al. 1980). The inactivation of thrombin by AT under normal physiological conditions is slow, but the rate of complex formation increases in a high degree by heparin cofactor activity (Machovich et al. 1976). AT has a therapeutic significance for those who suffer from venous thrombosis and pulmonary embolism with acquired or inherited inadequacy of AT activity (Beresford and Owen 1990). In these situations, an adequate supply of pure AT is essential.

Nevertheless, AT is available from human plasma (Büntemeyer et al. 1994; Munzert et al. 1997), from the view of product safety and possible virus infection (retrovirus and hepatotropic viruses), AT production must be only way through recombinant DNA (rDNA) technology (Zettlmeissl et al. 1987). AT has been produced using different expression systems like *E. coli*, Chinese hamster ovary cells and *Pichia pastoris*, COS 1 monkey kidney cells, BHK (baby hamster kidney) cells, *Spodoptera frugiperda* insect cells, and transgenic goat milk (Bock et al. 1982; Wasley et al. 1987; Zettlmeissl et al. 1988, 1989; Gillespie et al. 1991; Edmunds et al. 1998), but yeast-based recombinant human antithrombin (rhAT) has been demonstrated only by Broker et al. (1987).

To have the capacity to isolate a desired protein from a mixture, the physicochemical properties of the target protein should be used. Till now, there is no single or simple process to purify all kinds of proteins. On the other hand, if the protein is intended for clinical use, it ought to be very pure. But, as considered to be in economic view, purification should be done in a single step.

The main objective of a purification process is not only the exclusion of undesired contaminants, but also the purification of the target protein (Roe 2001). Ion exchange chromatography (IEC) is certainly the most commonly used technique for the separation and purification of proteins, polypeptides, nucleic acids, poly nucleotides, and other bio molecules. The explanations behind the accomplishment of IEC are its determining power, broad relevance, high capacity, and the effortlessness of the system (Bonnerjea et al. 1986). Size exclusion chromatography (SEC) is another purification process, in which components are filtered according to their size, and in some times molecular mass. It is frequently used to purify the larger or macro molecules such as proteins and industrial polymers (Paul-Dauphin et al. 2007).

In the present study, purification of rhAT from *Sacharomyces cerevisiae* BY4741 has been carried out using two different approaches such as cross flow filtration (CFF) and chromatography methods to achieve high recovery yields and purity. In addition, the effect of various physicochemical parameters such as mobile phase, mobile phase pH, flow rate, sample volume and sample concentration on rhAT purification were also investigated.

## Methods

### Chemicals and instrumentation

Tris or Tris aminomethane and 2-mercaptoetahnol were procured from Merck Millipore (USA). Fraction V or Bovine serum albumin (BSA) and phenylmethanesulfonylfluoride (PMSF) were obtained from Sigma (Germany). rhAT reference standard was purchased from NIBSC, UK. SDS-PAGE Mini-PROTEAN three apparatus was procured from Bio-Rad Laboratories Inc., (USA). Recombinant human AT was produced in a Minifors fermentor (Infors HT, Switzerland). The Fast protein liquid chromatography (FPLC) AKTA Prime Plus system, resins (stationary phase), bioprocess glass column (BPG XK16/20) for purification, cross flow filtration (CFF) system and the hollow fiber membrane cartridge (30 kDa) were purchased from GE Healthcare Life sciences (Uppsala, Sweden). The chromatographic data were recorded and analyzed by Prime View Evaluation™ software version 5. 31. Reversed phase high pressure liquid chromatography (RP-HPLC) equipped with UV/Vis (SPD 20A Prominence) detector was acquired from Shimadzu Corp. (Kyoto, Japan). The Jupiter 300 Å C4 LC column (250 × 4.6 mm) obtained from Phenomenex (USA).

### Production of rhAT

Large scale production of rhAT in *S. cerevisiae* BY4741 was performed in a fed-batch cultivation mode with a media volume of 3 L in Minifors HT bioreactor (Infors HT, Switzerland). The fermentation process was carried out in the following conditions: temperature 30 °C, pH 7.1, 40 % dissolved oxygen (DO). The foaming was controlled with a silicon antifoaming agent. The culture was induced with galactose (2 %) at an OD_600_ nm of 0.9 (dry cell weight = 11 g/L) of the fermented culture (Kim et al. 2003; El-Sayed et al. 1990).

### Cell harvesting and lysis

The fermented broth was harvested and the pellet was collected by centrifugation (Sorvall ST 16 R, Thermo scientific, Pittsburgh, PA, USA) at 4 °C and 40,000×*g* for 15 min. The pellet weight was calculated as dry cell weight (DCW, g/L). The cells were washed with wash buffer containing 0.05 M EDTA (pH 8.0) and 0.01 M Tris-HCl (pH 7.6). The cells were lysed by sonication (20 kHz, for 2–4 s time intervals) using lysis buffer containing 0.01 M Tris-HCl (pH 7.6), 1 % DMSO, 50 mM NaCl,1 mM EDTA, 0.5 % sucrose and 1 mM phenylmethylsulfonyl fluoride (PMSF).

### Concentration of rhAT protein solution

After cell lysis, the lysate was concentrated and simultaneously clarified using CFF system with a loading volume of 500 mL. The rhAT protein was filtered through 30 kDa molecular weight cutoff hollow fiber membrane cartridge with nominal flow path length of 30 cm with 650 cm^2^ of cross-sectional area. The maximum flux (flow) rate from the membrane was allowed at 10 mL/min. The filtration was accomplished using a stable pump speed at 300 rpm. The concentration intensity of the rhAT is distinguished by a concentration factor (*f*_C_) and retention factor (*R*_f_), and these are calculated from the following formula.

1 $$f_{\text{c}} = {{V_{\text{F}} } / {V_{\text{C}} }}$$

where *V*_F_ = feed volume and *V*_C_ = final cross (retentate) volume. The retention factor is also a significant parameter for the desired product characterization, and it is described as, the division of components between permeate and retentate.

2 $$R_{\text{f}} = {{({({C_{\text{F}} })^2 - C_{\text{p}} })} / {C_{\text{F}} }}$$

where, *C*_F_ = feed concentration and *C*_p_ = permeate concentration (Vemula et al. 2015).

### Purification of rhAT

Two different column chromatography techniques with different resins were selected to analyze the effect of stationary phase on rhAT purification. The principle of IEC with Q-Sepharose and Capto Q and CM Sepharose were applied to evaluate the effect of stationary phase on rhAT purification (Lutkemeyer et al. 1993). Simultaneously, the principles of SEC with three different resins (Sephacryl S200, Sephadex G-100 and Superose 12) were also applied to analyze the effect of stationary phase on yield and purity of rhAT (Štulík et al. 2003). A 25 mL of each stationary phase with 20 % ethanol solvent was filled through 0.2 Mpa pressure in the bioprocess glass (BPG) column (BPG XK16/20). The column was primarily washed with 100 mL of 0.5 M sodium chloride solution with a flow rate of 3.0 mL/min. The column is washed with 100 mL water to remove the salts and other contaminants during equilibration with the mobile phase. For every individual experiment, 2 mL of rhAT protein was loaded and the column pressure was maintained at 0.1–0.2 Mpa during the purification process. 50 mL of salt linear gradient program was run for the purifications from 20 to 100 % sodium chloride solution at a constant flow rate of 1.0 mL/min. Protein elution profiles were detected at 280 nm. All the purification experiments have been performed at 22 °C.

### Analytical procedures

The purity of the rhAT was assessed by reverse phase liquid chromatography (RP-HPLC) (Vanz et al. 2008) and SDS-PAGE (15 % w/v acrylamide (Faraji et al. 2010). The qualitative analysis of the rhAT was analyzed through RP-HPLC. HPLC system (Shimadzu, Kyoto, Japan) was equipped with LC Prominence SPD 20A UV–Visible detector and LC 20 AT binary pumps. The rhenodyne type of injector port was used to inject the samples. The rhAT analysis was carried out at the following conditions: flow rate 1.0 mL/min, wavelength 280 nm, pressure 80 kgf and controlled temperature 40 °C. The Jupiter 300 Å C4 5 µ column (250 × 4.6 mm) (Phenomenex, USA) was used to analyze the samples. The mobile phase A consisted of water: acetonitrile (87.5:12.5 v/v) and mobile phase B consisted of water: acetonitrile (30:70 v/v). Mobile phase A and B consisted of TFA (ion pairing agent) with the concentration of 0.1 and 0.08 %, respectively. The rhAT was eluted using linear gradient mode with % B to 90 % B in 20 min and 90 % B to 5 % B in 40 min. The purified rhAT concentration was calculated by Bradford assay using BSA as a standard (Tesio et al. 2011).

On the other hand, Purified rhAT III and control was loaded onto 15 % SDS-PAGE and transferred to polyvinylidene difluoride membrane and western blot analysis was performed using polyclonal antibody generated from rabbits and Horseradish peroxidase conjugated goat IgG antibody (Bio-Rad Inc, USA) (Kuwae et al. 2005). The biological potency of the purified rhAT III was measured as heparin cofactor (HC) activity with a commercial assay kit that uses the thrombin-specific chromogenic substrate S-2238 (TESTZYM ATIII kit, Daiichi Pure Chemical, Tokyo).

## Results and discussion

### Preparation of concentrated solution and fractional purification of rhAT

**Fig. 1:**
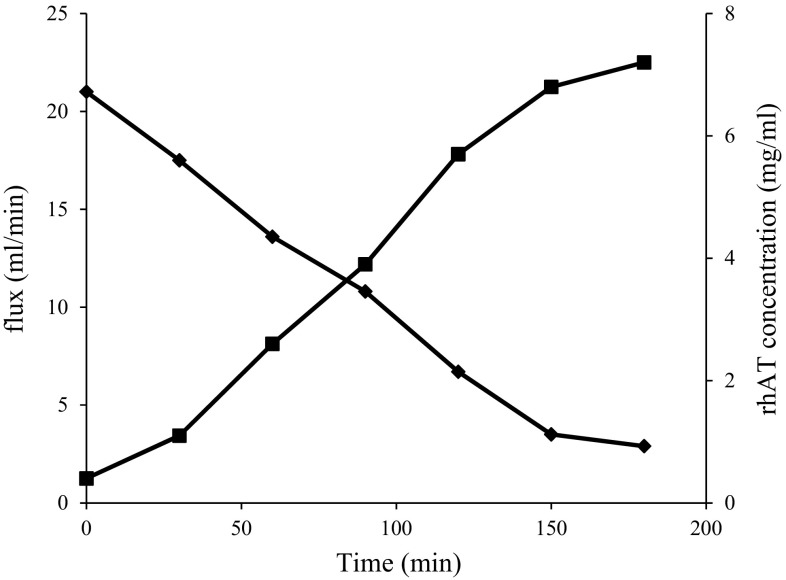
Clarification and concentration of rhAT. Effect of filtration during ultra filtration with hollow fiber cartridge, rhAT concentration was shown increasing with respective to the decrease in flux as a function of time; *filled square* represents rhAT concentration; *filled diamond* represents flux

Cell lysate was concentrated and partially purified by cross flow filtration system using 30 kDa catridge. The rhAT filtration performance is shown in Fig. 1. From the experimental results, the change in rhAT concentrations (0.4, 1.1, 2.6, 3.9, 5.7, 6.8 and 7.2 mg/mL) with change in flux (21, 17.5, 13.6, 10.8, 6.7, 3.5 and 2.9) was observed (Fig. 1) at progressive time intervals (0, 30, 60, 90, 120, 150 and 180 min). It can be clearly observed that a noteworthy increase in protein concentration has been observed with decrease in flux as a function of time. At a specific time period (180 min), no significant change was observed and a high 7.2 mg/mL of rhAT concentration was obtained with a constant flux of 2.9 mL/min. This effect may be due to the clogging of pores by formation of foam on the surroundings of membrane that constrained the filtration of solution from the membrane. The filtration was ended after recovering 40 mL retentate with a concentration factor of 12.5 and retention factor of 0.87. The rhAT recovery yield and purity after the filtration step was about 87 and 94 %, respectively. Various recombinant proteins such as recombinant human granulocyte stimulating factor (rhG-CSF) and monoclonal antibodies have been successfully concentrated with cross flow filtration system and it was achieved with >95 % purity (Vemula et al. 2015; Liu et al. 2010).

### Effect of chromatographic resin on rhAT purification

Optimization should evaluate a number of physicochemical parameters including stationary and mobile phase for effective protein purification. Therefore, the studies have been conducted on rhAT purification to analyze the influence of ion exchange and size exclusion chromatographic resins. The recovery yield and purity of rhAT for both ion exchange and size exclusion chromatography with different resins were shown in Table 1. From the results, it can be seen that among various resins selected in both ion exchange and size exclusion chromatography, size exclusion chromatography with Superose 12 showed maximum recovery yield and purity of 51 and 97 %, respectively. The lower yields with ion exchange could be attributed to low permeability and capacities of resins for larger proteins such as antithrombin. Additionally, the hydrophobic character of the ion exchange resin will denature the proteins (Janson 2012). Therefore, further experiments were carried out with Superose 12. The above experimental results obtained in the present work were significantly higher than the work reported by Mak et al. (1996).The effect of various chromatographic resins on recombinant protein (rhG-CSF) purification was also successfully studied by other researchers (Wang et al. 2006).

**Table 1:** Effect of stationary phase on rhAT purification, the maximum yield (51 %) and purity (97 %) of rhAT was shown with Superose 12. The results represent mean values with standard deviation (SD) of three repeated (*triplicates*) experiments

	Ion exchange resin			Size exclusion resin		
	Q-Sephrose	Capto Q	CM Sepharose FF	Sephacryl S200	Sephadex G-100	Superose 12
Recovery yield (%)	43 ± 1.2	46 ± 1.2	48 ± 1.1	49 ± 1.2	48 ± 1.2	51 ± 1.1
Purity (%)	96	96	97	97	97	97

### Effect of Mobile phase on rhAT purification

The choice of mobile phase plays a critical role in the purification of proteins because they ought to be adapted to the buffer atmosphere during the elution. In the present work, a study has been performed on four different mobile phases (tris, acetate, phosphate and sulfate) with pH 7.2 using size exclusion chromatography with Superose 12. The recovery yield and purity of rhAT with four different mobile phase buffers were shown in Table 2. From the experimental results, recovery yields (50, 52, 54 and 51 %) and purities (97, 98, 98 and 97 %) of rhAT were obtained with respective mobile phase buffers such as tris, acetate, phosphate and sulfate buffers. From the above result analysis, it was concluded that phosphate buffer has achieved a high recovery yield (54 %) with 98 % purity. The lowest recovery yield (50 %) was achieved with Tris buffer with 97 % purity. However, acetate and sulfate are next to the phosphate with recovery yields of 52 and 51 %, respectively. The results attained in the present work were higher than the previously reported work (Mochizuki et al. 2001).The effect of mobile phase on recombinant protein (rhG-CSF) purification was also successfully studied by other researchers (Vemula et al. 2015).

**Table 2:** Effect of mobile phase on rhAT purification. The maximum recovery yield (54 %) and purity (98 %) of rhAT was shown with Superose 12 matrix. The results represent mean values with standard deviation (SD) of three repeated (triplicates) experiments

Mobile phase buffer	Recovery yield (%)	Purity (%)
Tris	50 ± 1.1	97
Acetate	52 ± 1.2	98
Phosphate	54 ± 1.1	98
Sulfate	51 ± 1.1	97

### Effect of mobile phase pH on rhAT purification

**Fig. 2:**
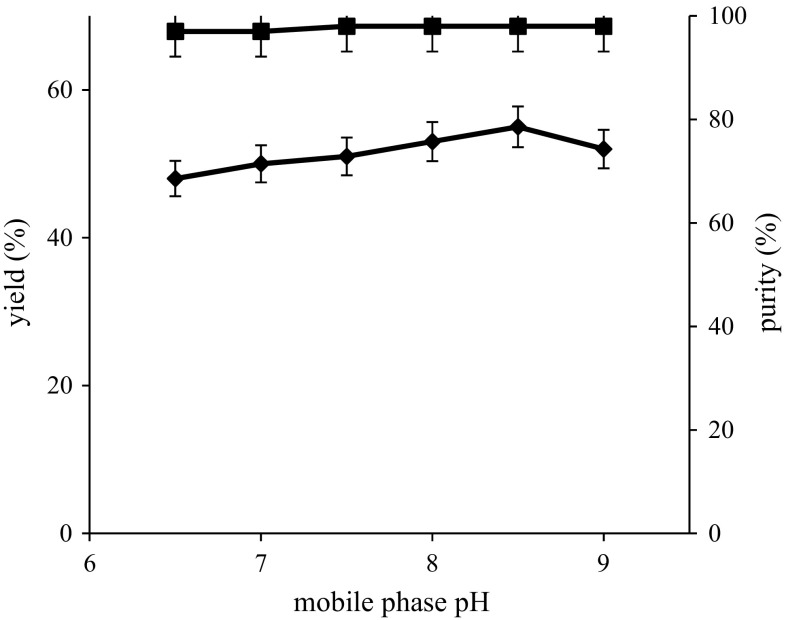
Effect of mobile pH on rhAT purification, the maximum yield (55 %) and purity (98 %) of rhAT was shown with pH 8.5. *Filled diamond* represents recovery yield; *Filled square* represents purity. The results represent mean values with standard deviation (SD) of two repeated (*duplicates*) experiments

Purification of proteins is pH dependent, which can definitely affect the recovery yield and purity (Burgess et al. 1987). In the present work, a study has been carried out on phosphate buffer with different pH values ranging from 6.5 to 9 to estimate their effect on recovery yield and purity of rhAT. The recovery yield and purity of rhAT at various phosphate buffer pH values were shown in Fig. 2. From the experimental results, recovery yields (48, 50, 51, 53, 55 and 52 %) and purities (97, 97, 98, 98, 98 and 98 %) of rhAT were obtained with respective mobile phase pH such as 6.5, 7, 7.5, 8, 8.5 and 9. It has been clearly observed that the maximum recovery yield (55 %) and purity (98 %) of rhAT was observed with buffer at pH 8.5. The lowest recovery yield (48 %) and purity (97 %) of rhAT was observed with buffer at pH 6.5. Even though the purity was high (98 %) at pH 7.5,8 and 9, low recovery yields (51, 53 and 52 %) were observed when compared to pH 8.5.The mobile phase pH on recombinant protein purification was also successfully analyzed by previously reported work (Vemula et al. 2015).

### Effect of flow rate on rhAT purification

**Fig. 3:**
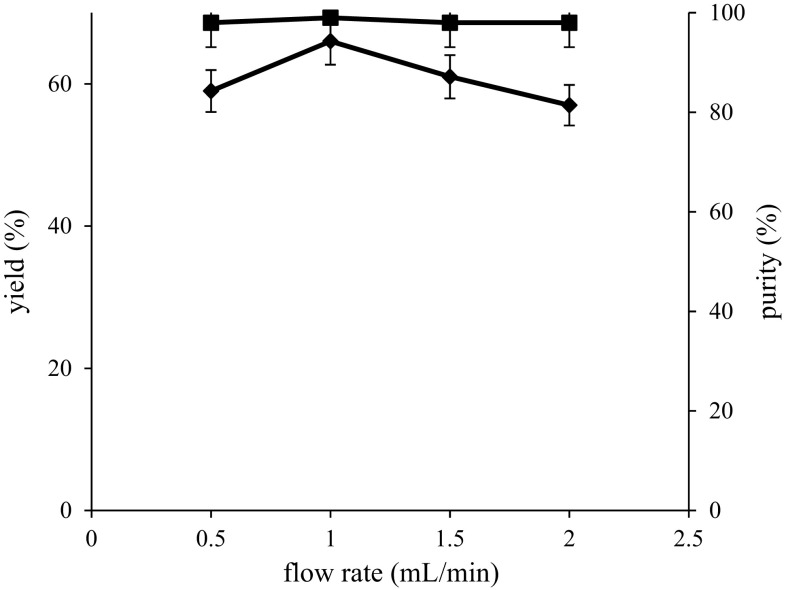
Effect of flow rate on rhAT purification. The maximum yield (66 %) and purity (99 %) of rhAT was shown with 1 mL/min flow rate. *Filled diamond* represents recovery yield; *filled square* represents purity. The results represent mean values with standard deviation (SD) of two repeated (*duplicates*) experiments

Flow rate should be considered as one of the most significant parameters during protein purification, because in size-based separations, it may influence the protein structure, biological activity and recovery yield. In the present work, a study has been conducted with different flow rates of 0.5, 1.0, 1.5 and 2.0 mL/min to show the impact of flow rate on rhAT purification. The recovery yield and purity of rhAT at different flow rates were shown in Fig. 3. From the experimental results, recovery yields (59, 66, 61 and 57 %) and purities (98, 99, 98 and 98 %) of rhAT were obtained with respective flow rates such as 0.5, 1, 1.5 and 2 mL/min flow rate. It can be clearly seen that the high recovery yield (66 %) and purity (99 %) of rhAT was obtained with 1 mL/min flow rate. Although using lower sample flow rates results in longer run times, the increased resolution gives greater confidence and optimum column efficiency (Oliva et al. 2001). The low recovery yield (57 %) was observed with 2 ml/min, due to high acceleration, which increased the chance of escaping and decreased the passage of the sample from the resin resulting in low recovery yields. Though the purity of rhAT is similar at 0.5, 1.5 and 2 flow rates, variations in recovery yields (59, 61 and 57 %) were observed. Hence further experiments were carried out with 1.0 mL/min flow rate. The results obtained in this study were very close to the previously reported work (Wang et al. 2014)

### Effect of sample volume on rhAT purification

**Fig. 4:**
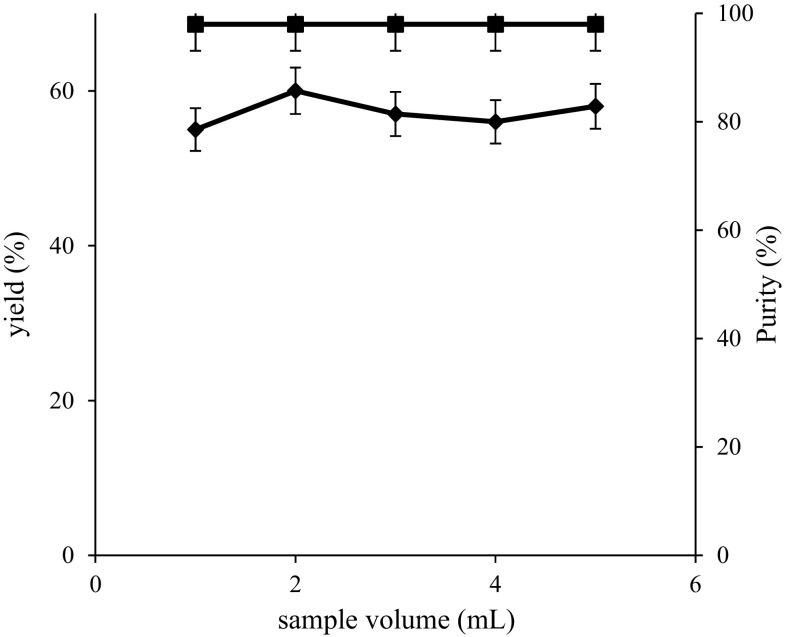
Effect of sample volume on rhAT purification. The maximum recovery yield (60 %) and purity (98 %) of rhAT was achieved with 2 mL sample volume. *Filled diamond* represents recovery yield; *filled square* represents purity. The results represent mean values with standard deviation (SD) of two repeated (*duplicates*) experiments

Sample injection volume can influence the protein purification and affects mostly on recovery yields. Therefore, in the present study, several experiments were carried out on rhAT purification with different sample volumes such as 1, 2, 3, 4 and 5 mL. The recovery yield and purity profiles of rhAT with varying sample volumes were shown in Fig. 4. From the experimental results, recovery yields (55, 60, 57, 56 and 58 %) and purities (98, 98, 98, 98 and 98 %) of rhAT were obtained with respective sample volumes such as 1, 2, 3, 4 and 5 mL. From result analysis, it was concluded that the maximum recovery yield (60 %) and purity (98 %) with the sample volume of 2 mL were observed. This is attributed to the suitable volume of the sample that enters through the resin and increases the passage of desired protein molecules. The lowest recovery yields of 55 and 56 % were achieved with 1 and 4 mL volumes, respectively. This is due to broadening of sample zone by increasing the sample volume and decreased the passage of the sample through the resin. Therefore, further studies were continued with 2 mL sample volume.

### Effect of protein concentration on rhAT purification

**Fig. 5:**
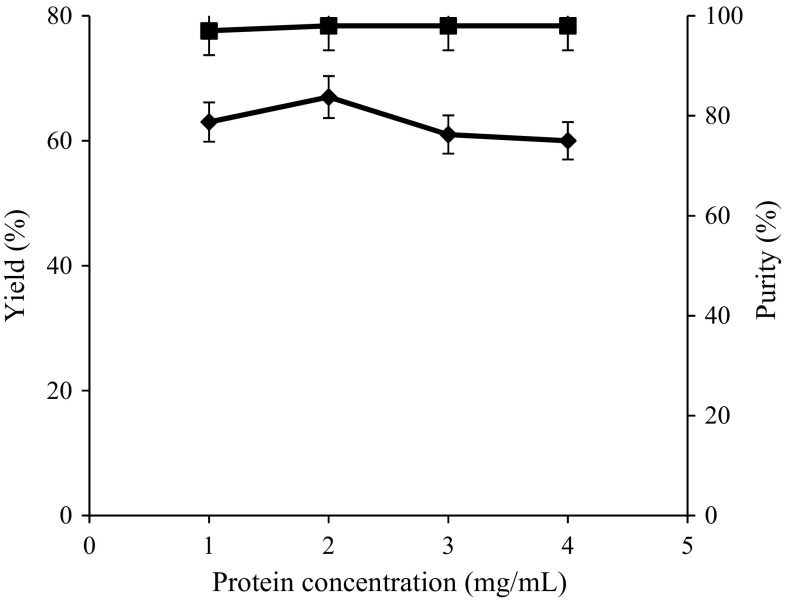
Effect of protein concentration on rhAT purification. The maximum yield (67 %) and purity (99 %) of rhAT was shown with 2 mg/mL concentration. *Filled diamond* represents recovery yield; *filled square* represents purity. The results represent mean values with standard deviation (SD) of two repeated (*duplicates*) experiments

Sample concentration may also influence the chromatographic resolution and sensitivity (Hong et al. 2012). In the present work, a study has been conducted on different protein concentrations ranging from 1 to 4 mg/ml with Superose 12 matrix to analyze the protein concentration effect on recovery yield and purity of rhAT. Figure 5 shows the recovery and purity of rhAT under various sample concentrations. From the experimental results, recovery yields (63, 67, 61 and 60 %) and purities (98, 99, 99 and 98 %) of rhAT were obtained with respective protein concentrations such as 1, 2, 3 and 4 mg/mL. From the above result analysis, it can be clearly seen that the high recovery yield (67 %) and purity (98 %) of rhAT were achieved with protein concentration of 2 mg/mL. Even though a high purity (98 %) of rhAT was observed with protein concentration of 3 mg/mL, recorded low recovery yields of 61 %. From this study, it was concluded that the loss of recovery yields could be observed with increase in protein concentration due to non-binding interactions of protein with resin.

### Optimal conditions for rhAT purification

**Fig. 6:**
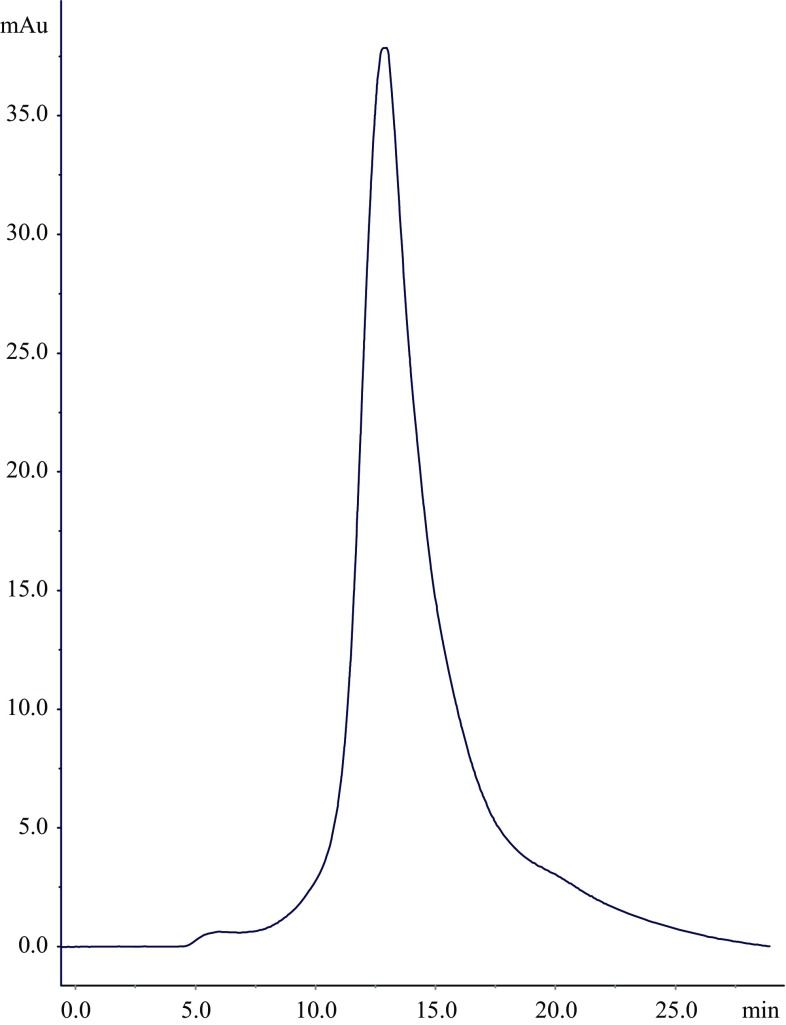
FPLC chromatogram of rhAT eluted from the SEC column. The maximum yield (67 %) and purity (99 %) of rhAT was shown under optimum conditions: stationary phase: Superose 12; phosphate buffer with pH: 8.5; flow rate: 1 mL/min; sample volume: 2 mL and sample concentration: 2 mg/mL

**Fig. 7:**
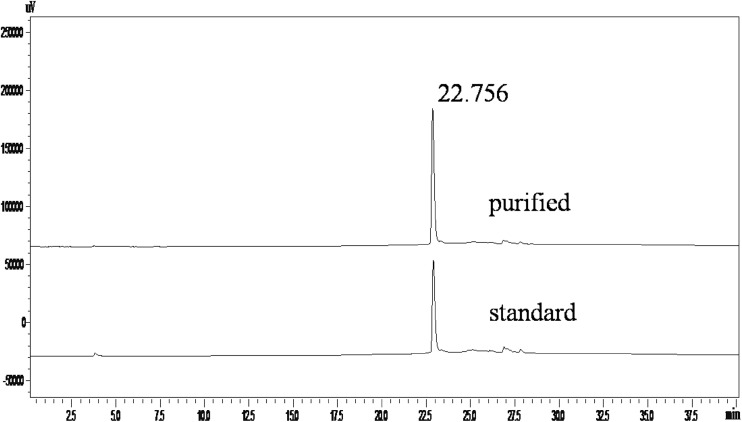
RP-HPLC analysis of rhAT, the purity of the rhAT was shown to be ≥98 %

Based on previous experiments, the optimal chromatographic conditions were preferred for rhAT purification: Superose 12 matrix, phosphate buffer at pH 8.5, flow rate 1 mL/min, sample volume 2 mL and sample concentration is 2 mg/mL. Figure 6 shows the FPLC chromatogram of rhAT eluted under the aforesaid optimized conditions. It can be observed that the single major peak was observed and indicates the monomeric form of rhAT. The major peak appeared with high resolution at a retention volume of 13.3 mL indicating the active fraction of rhAT. The whole chromatographic process was carried out in 25 min. The obtained recovery yield and purity of rhAT under optimum conditions were 67 % and ≥98 %. The above experimental results obtained in the present work were significantly higher than the previously reported work (Büntemeyer et al. 1994; Mochizuki et al. 2001) and obtained results were also very close to the earlier reported work (Wang et al. 2014). Figure 7 shows the RP-HPLC chromatogram eluted from the SEC column under optimized conditions. It can be seen that the purity of rhAT was shown to be 98 as similar with retention time (22.756 min) of standard rhAT.

**Fig. 8:**
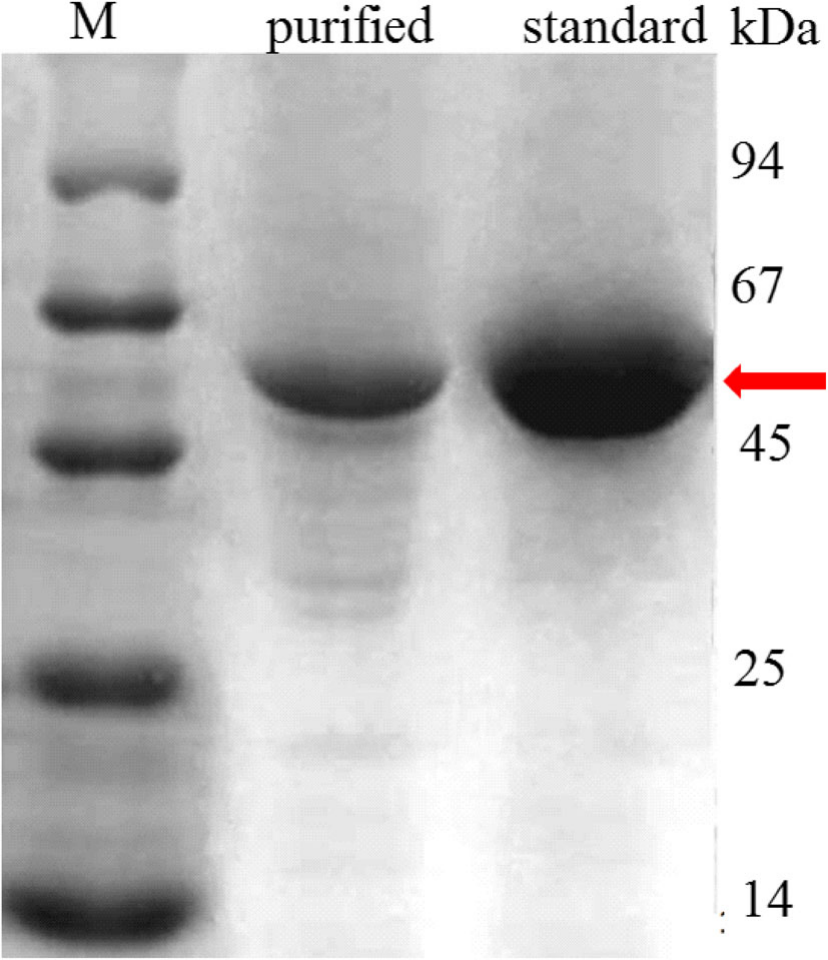
SDS-PAGE analysis of rhAT: *Lane M* marker; *Lane 2* purified rhAT; *Lane 3* reference standard. A single target band was observed with molecular weight of approximately 58KDa, which is similar to the reference standard

**Fig. 9:**
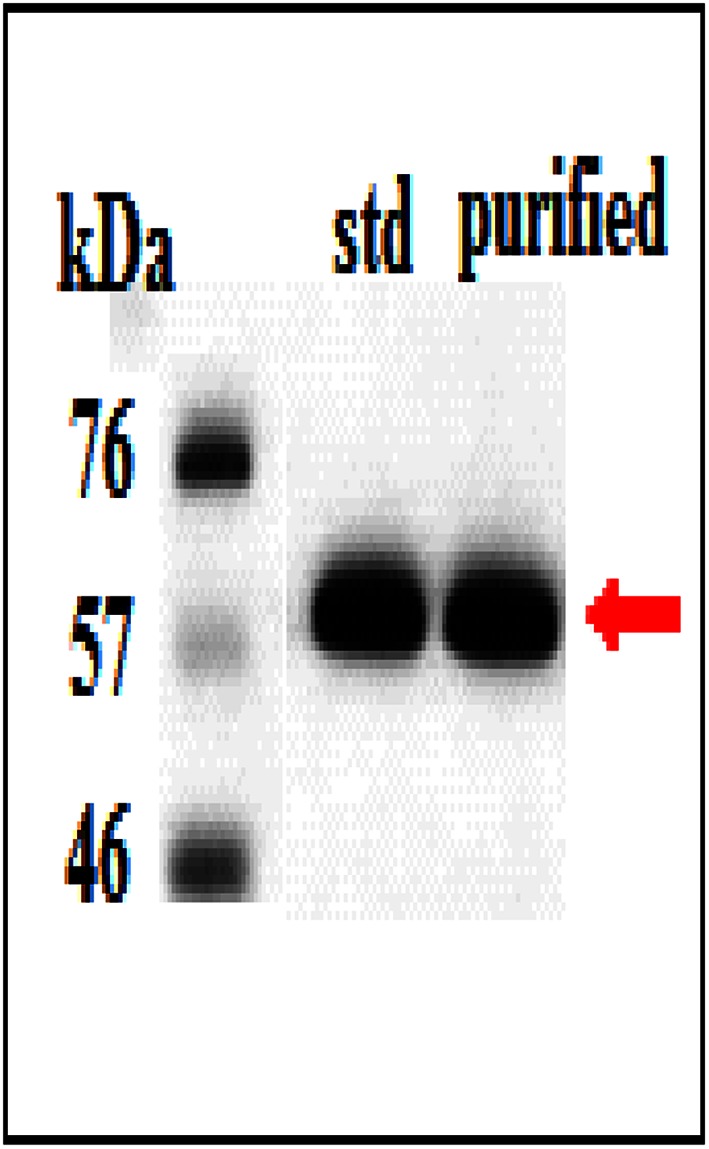
Western blot analysis of rhAT. Both purified and reference standard rhAT bands were characterized using polyclonal antibody and HRPO conjugated goat IgG antibodies against antithrombin

The purity of rhAT was also assessed through SDS-PAGE and western blotting. SDS–PAGE analysis of rhAT III was shown in Fig. 8. A single target rhAT III band confirms the molecular mass of 58 kDa, which is similar to the control (standard rhAT III). On the other hand, gel was transferred to PVDF membrane and the transformation was immuno detected with rhAT III antibodies and a single rhAT III band was shown in Fig. 9, which is analogous to the reference standard. Finally, biological activity of purified rhAT was found to be 410 IU/L measured as heparin cofactor (HC) activity.

## Conclusions

In summary, SEC is found to be an effective technique for rhAT purification and showed a maximum recovery yield (51 %) and purity (97 %) when compared to the IEC recovery yield (48 %) and purity (97 %). Further investigations on physicochemical chromatographic conditions such as mobile phase, mobile phase pH, flow rate, sample volume and sample concentration were also proved their influence on recovery yield and purity. Under the optimized conditions, the maximum recovery yield, purity and biological activity of rhAT was found to be 67, 99 % and 410 IU/L, respectively. The purity was evaluated through various analytical techniques such as RP-HPLC, SDS-PAGE and western blotting.
